# MXene-Based Nanomaterials for Multifunctional Applications

**DOI:** 10.3390/ma16031138

**Published:** 2023-01-29

**Authors:** A.A.P.R. Perera, K.A.U. Madhushani, Buwanila T. Punchihewa, Anuj Kumar, Ram K. Gupta

**Affiliations:** 1 Department of Chemistry, Pittsburg State University, Pittsburg, KS 66762, USA; 2 National Institute for Materials Advancement, Pittsburg State University, Pittsburg, KS 66762, USA; 3 Department of Chemistry, University of Missouri-Kansas City, Kansas City, MO 64110, USA; 4 Nano-Technology Research Laboratory, Department of Chemistry, GLA University, Mathura 281406, Uttar Pradesh, India

**Keywords:** MXenes, MXene composites, energy storage, electrocatalysts, sensors

## Abstract

MXene is becoming a “rising star” material due to its versatility for a wide portfolio of applications, including electrochemical energy storage devices, electrocatalysis, sensors, biomedical applications, membranes, flexible and wearable devices, etc. As these applications promote increased interest in MXene research, summarizing the latest findings on this family of materials will help inform the scientific community. In this review, we first discuss the rapid evolutionary change in MXenes from the first reported M_2_XT_x_ structure to the last reported M_5_X_4_T_x_ structure. The use of systematically modified synthesis routes, such as foreign atom intercalation, tuning precursor chemistry, etc., will be further discussed in the next section. Then, we review the applications of MXenes and their composites/hybrids for rapidly growing applications such as batteries, supercapacitors, electrocatalysts, sensors, biomedical, electromagnetic interference shielding, membranes, and flexible and wearable devices. More importantly, we notice that its excellent metallic conductivity with its hydrophilic nature distinguishes MXene from other materials, and its properties and applications can be further modified by surface functionalization. MXene composites/hybrids outperform pristine MXenes in many applications. In addition, a summary of the latest findings using MXene-based materials to overcome application-specific drawbacks is provided in the last few sections. We hope that the information provided in this review will help integrate lab-scale findings into commercially viable products.

## 1. Introduction

Presently, because of innovations in science and nanotechnology, the knowledge and applications of nanomaterials and nanomaterial-based composites are constantly changing. The unique size and composition-specific properties of nanomaterials help solve many challenges in science. Nanomaterials can be classified based on their morphology, dimensionality, size, agglomeration state, and composition, which, depending on each characteristic, makes them suitable for a wide variety of applications. Two-dimensional (2D) nanomaterials have an atomic thickness, ample active surface sites, a large surface area-to-volume ratio, and excellent mechanical properties, which make them ideal for multiple applications, most prominently in energy storage, electronics, sensors, catalysis, and biomedical applications [1,2]. In that sense, it is further confirmed that the class of 2D nanomaterials is one of the most prominent and widely used/studied materials so far. Graphene, the first form of 2D nanomaterial, was discovered in 2004 by Novoselov et al. [2]. Subsequently, there have been numerous 2D nanomaterials, including, for instance, hexagonal boron nitride, transition metal dichalcogenides, and phosphorene. Among this list, graphene’s unique honeycomb-like single-atomic structure makes it highly conductive and stronger [3].

Although graphene has made more significant progress in science and technology than all other 2D materials, its simple structure and chemistry limit its further advancement. Recently, a group of new materials labeled MXenes has significantly influenced inventive ideas in each field of scientific research. Its complex atomic arrangement and multilayered structure with excessive tunable properties enhance its multifunctional behaviors. Examples are the breakthroughs made in energy storage devices, catalysts, sensors, antennas and RFID tags, biomedical applications, electromagnetic interference shielding (EMI), nanocomposite-hybrid materials, environmental and water purification, etc. [4]. With the emerging applications of MXene in various fields, an idea has arisen among the scientific community that “MXenes are the future of nanotechnology”. In new research studies, the ability to tune the surface, electrical and electrochemical properties by tailoring the surface functional groups of MXene, as in graphene, further supports this notion. Most MXenes and MXene-based materials have high volumetric capacitance, antibacterial properties, electrochromic behavior, high electronic conductivity, and optical transparency [4]. Therefore, MXenes open the door to new applications and modify/improve the performance of current applications. 

**Figure 1:**
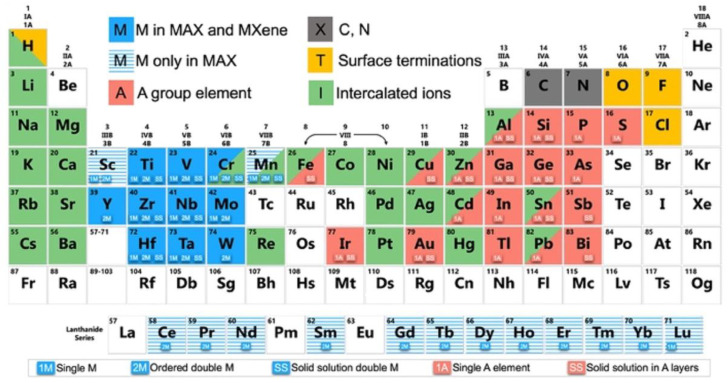
Elements used to build MAX phases, MXenes, and their intercalated ions. Adapted with permission [9]. Copyright 2019, American Chemical Society.

For the past 11 years, starting with the discovery of the first MXene in 2011, many academic researchers have led experiments using a variety of compositions and structures of MXenes. According to the literature, the MXene family includes carbides, nitrides, and carbonitrides with the structure of M_n+1_X_n_T_x_, where M is an early transition metal (Ti, V, Mo, Ta, etc.), X is C and/or N, T_x_ represents the surface groups (typically =O, −OH, −F, and −Cl), and *n* = 1–4. MXenes are produced by the selective chemical etching of specific atomic planes from layered carbide/nitride precursors known as MAX phases. Generally, MAX-phase carbide/nitride precursors consist of an M_n+1_AX_n_ chemical formula, where A represents Al or Si. As shown in Figure 1, depending on the composition of the transition elements in the MAX phase, 2–5 atomic layers of the transition metal may exist in the MXene. Every n layers of M atoms are interleaved with layers of pure A; the X atoms occupy the octahedral sites between the M atoms [5,6].

**Figure 2:**
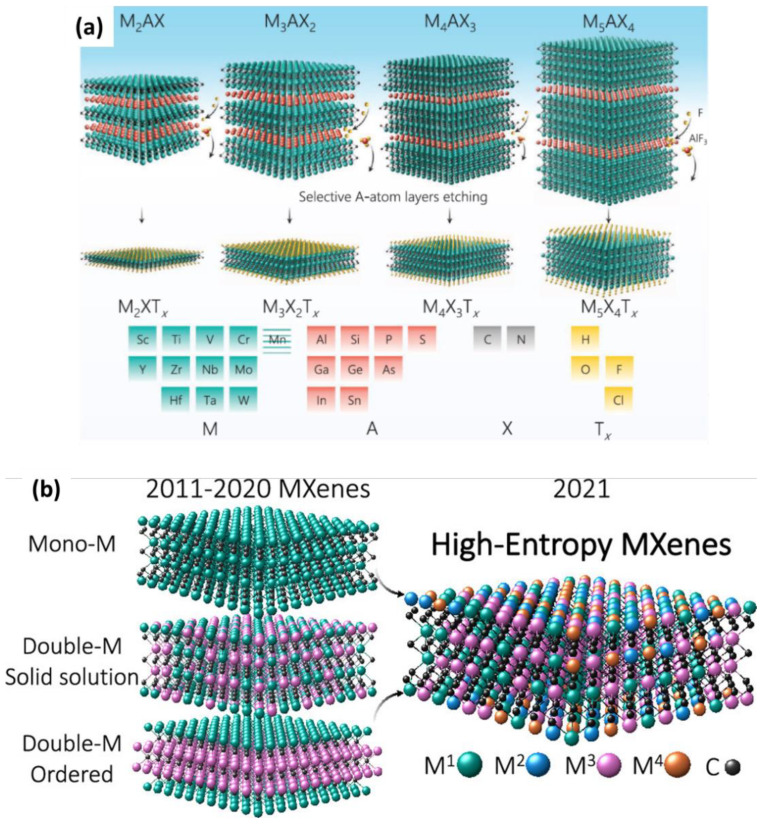
The HF-based synthesis of MXene from its MAX precursor and its effects as well as various forms of MXene. (**a**) A schematic representation of MAX structures from *n* = 1 to *n* = 4 and their etched MXene structures with transition metals, carbon/nitrogen, the majority of A-group elements, and surface terminations. Adapted with permission [10]. Copyright 2021, John Wiley and Sons. (**b**) High-entropy MXenes. Adapted with permission [11]. Copyright 2021, American Chemical Society.

To date, four different compositions of Mxenes have been synthesized: M_2_XT_x_, M_3_X_2_T_x_, M_4_X_3_T_x_, and M_5_X_4_T_x_ (Figure 2a). According to their structure, they can be identified as mono-transitional metal (TM) MXenes, double-TM solid solution MXenes, double-TM ordered MXenes and high-entropy MXenes. The recently discovered high-entropy MXenes, as mentioned in Figure 2b, have added great diversity to the MXene family. According to Dadashi et al., more than 40 MXene structures were reported by 2021, and theoretically, more than 100 possible compositions of MXenes have been predicted to date. Figure 1 shows elements that can be used experimentally and theoretically for the synthesis of MXenes and MAX phases [5,7,8,9]. In this review, the future of MXenes and their potential impact on energy storage, electrocatalysis, sensors, biomedical applications, and other emerging applications are discussed in depth.

## 2. Methods to Synthesize MXene

MXene has been shown to have intrinsic properties based on its composition and structure. Therefore, the structures and synthesis routes of previously successfully produced MXenes are discussed throughout this section. Furthermore, this section provides a scientific understanding of the production of the most studied and widely used MXene materials safely and reliably.

### 2.1. Pure HF-Based Synthesis Routes

**Figure 3:**
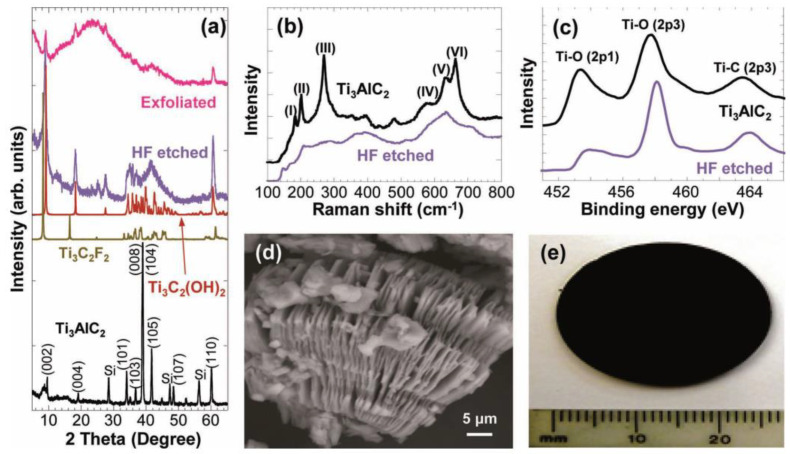
(**a**) XRD pattern, (**b**) Raman spectra, (**c**) XPS spectra of the Ti_3_AlC_2_ before and after exfoliation. (**d**) SEM image and (**e**) Cold-pressed disk of etched and exfoliated material after HF treatment. Reproduced with permission [7]. Copyright 2014, John Wiley and Sons.

Many procedures for the synthesis of MXenes are based on the top-down method, in which exfoliation of the layered transition metal carbide, Ti_3_AlC_2_, occurs by immersing Ti_3_AlC_2_ powders in 50% hydrofluoric acid (HF). During the exfoliation process, Al layers are selectively etched and replaced by −OH, and −F surface groups. After the etching process, the Ti_3_C_2_T_x_ 2D layers are held together by hydrogen and van der Waals bonds. The suspension was centrifuged at 3500 rpm and washed several times using deionized water to neutralize the HF (etchant). The Ti_3_C_2_T_x_ was separated into multilayered (ML) powder form. The XRD spectra, Raman spectra, and XPS spectra of Ti_3_AlC_2_ before and after HF treatment are shown in Figure 3, which confirms the successful exfoliation into MXene. The SEM image of the sample after HF treatment is similar to exfoliated graphite, which provides a high surface-area-to-volume ratio. Most importantly, the metallically conducting and hydrophilic behavior of MXenes was found to be remarkable compared to graphene; therefore, these materials are considered for usage as multifunctional materials, such as in catalysis, energy storage/pseudocapacitors, and Li-ion batteries [7]. 

In a similar study, Ti_2_AlC, Ta_4_AlC_3_, (Ti_0.5_,Nb_0.5_)_2_AlC, (V_0.5_,Cr_0.5_)_3_AlC_2_, and Ti_3_AlCN MAX phases were exfoliated into Ti_2_C, Ta_4_C_3_, TiNbC, (V_0.5_,Cr_0.5_)_3_C_2_, and Ti_3_CN_x_ MXene powders at room temperature. Therefore, many chemically diverse, Al-containing MAX phases can be exfoliated using the HF synthesis route. Similar to Ti_3_AlC_2_, layered hexagonal ternary metal carbides and nitrides of Ti, V, Cr, Nb, Ta, Hf, Zr, and Mo belong to the MAX phases and can be exfoliated into various types of MXenes via the following chemical reactions [8].

M_n+1_AlX_n_ + 3HF → M_n_+1X_n_ + AlF_3_ + 1.5H_2_ (1)

M_n+1_X_n_ + 2H_2_O → M_n+1_X_n_ (OH)_2_ + H_2_ (2)

M_n+1_X_n_ + 2HF → M_n+1_X_n_F_2_ + H_2_ (3)

**Figure 4:**
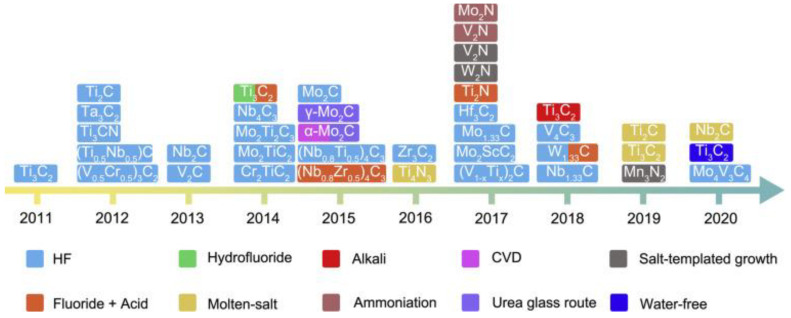
Timeline of a typical synthetic route for MXene in the past decade. Adapted with permission [13]. Copyright 2021, Elsevier.

Following this, they reported the chemical intercalation of surface-functionalized Ti_3_C_2_ with an intercalant such as urea, hydrazine monohydrate (N_2_H_4_. H_2_O), N, N-dimethylformamide (DMF), and dimethyl sulphoxide (DMSO). This facilitated the delamination of stacked Ti_3_C_2_ layers into separate 2D nanosheets in the solvent (colloidal solution of MXene) after sonication. The results of this study concluded that the intercalation of MXene layers would be an attractive synthetic route to achieve high capacitance and high cycling rates of MXene-based anodes in energy storage devices [8]. Later, this phenomenon was confirmed by several studies. Thus, the interlayer electron-transfer process of MXene is sensitive to the spacing distance between neighboring layers, which varies regarding temperature, cation intercalation, and flake morphology [12]. Scientists have further modified HF-based synthesis routes to produce numerous MXene-based compounds in less hazardous and cost-effective ways. Chemical etching via in situ HF and mixed acids are the leading techniques among these. Figure 4 shows the timeline of a typical synthetic route for MXene in the past decade. 

### 2.2. Mixed HF-Based Synthesis Routes

To date, thin films of MXenes have attracted considerable attention because of their large applications in the field of electronics, photonics, and sensing applications for preparing transparent conductive electrodes. Firstly, Halim et al. studied the feasibility of the preparation of epitaxial Ti_3_C_2_T_x_ films by the room temperature selective etching of Al from sputter-deposited epitaxial Ti_3_AlC_2_ thin films using HF and ammonium bifluoride (NH_4_HF_2_) etchants. The NH_4_HF_2_ etching process leads to the simultaneous intercalation of NH_3_ and NH_4_^+^ species, which provides larger c-lattice parameters (~25 Å) than films etched with HF. The c-lattice parameter value corresponds to the interlayer space among MXene films. The intercalated Ti_3_C_2_T_x_ films have exhibited higher transparencies with excellent metallic conductivity than their Ti_3_C_2_T_x_ counterparts. In addition, the other advantage of NH_4_HF_2_ is that it is a mild etchant and less hazardous than HF, and it can be a good substitute for hazardous HF. It is thus confirmed that the NH_4_HF_2_ etching is a potential pathway for the synthesis of the MXene-based transparent conductive thin films [14].

By the reaction of the MAX phase with HF, (=O), (−OH), and (−F) functionalities are introduced in the MXene structure, which expands the layer separation and makes the material hydrophilic. The fluoride salt etching route enables the MXene to be intercalated in the exfoliation process. As per the previous literature, a highly hazardous HF synthesis route can potentially substitute the HF-based synthesis routes. For example, Ghidiu et al. proposed a new synthesis route to prepare clay-like Ti_3_C_2_ MXene by etching Al from the Ti_3_AlC_2_ MAX phase using lithium fluoride (LiF) and hydrochloric acid (HCl) solution. The resultant clay-like material can be dried and shaped into a highly conductive solid or rolled into micrometer-thin films. Fluoride salts, such as NaF, KF, CsF, tetrabutylammonium fluoride, and CaF_2_ in HCl or H_2_SO_4_, also showed similar etching behavior to LiF. IN particular, this single-step etching and intercalation process enhances the volumetric capacitances, with excellent cyclability and rate performances of MXene-based electrodes compared to the MXene-based electrodes prepared by the conventional HF synthesis route [15]. Based on this synthetic method, various fluoride salt etching routes have been modified during the past years. A study proposed the selective etching of Al from Ti_3_AlC_2_ and Ti_2_AlC, similar to those reported previously in the literature, using a mixture of iron fluoride (FeF_3_) and HCl. Iron (Fe) is the fourth most abundant element in the Earth’s crust, and the FeF_3_ + HCl mixture is less expensive and safer than working with HF. Furthermore, the etching process of FeF_3_ + HCl can significantly alter the hydrophilic properties of MXene by controlling the surface functionalization. This offers new opportunities to fabricate high-performance materials based on MXenes for catalytic, electrocatalytic, and capacitance applications [16]. 

Another efficient method to synthesize MXene is the mixed-acid method. In 2018, a neuroelectronic device was constructed by a high-throughput microfabrication process using Ti_3_C_2_ MXene microelectrodes. The device exhibits superior impedance and in vivo neural recording performance compared to standard metal microelectrodes. They synthesized Ti_3_C_2_ MXene by selectively etching atomic layers of aluminum from Ti_3_AlC_2_ in an aqueous HF and HCl solution for 24 h. The notable difference was that manual agitation with a mixed acids system was carried out to create a homogeneous dispersion of large Ti_3_C_2_ flakes that stimulate conductivity [17]. An additional subfamily of M_5_X_4_T_x_ MXenes with five layers of TM was found in 2019. As the first M_5_X_4_T_x_ MXene, Mo_4_VC_4_T_x_ was synthesized with a phase-pure Mo_4_VAlC_4_ MAX phase by the top-down method. Due to their higher thickness, M_5_X_4_T_x_ MXenes could have the potential to be useful in many applications, including, but not limited to, structural materials, optoelectronic devices with high figures of merit, and electronics. Additionally, the Mo_4_VAlC_4_ MAX phase exhibits twinning on the center TM layers of atoms, which makes Mo_4_VC_4_ MXene different from all other known MXenes [4].

**Figure 5:**
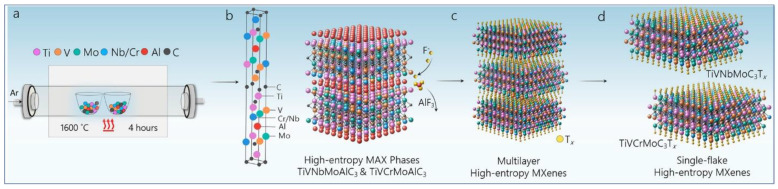
Schematic for the synthesis of MAX and MXenes. (**a**) Reactive sintering of high-entropy MAX phases. (**b**) MAX phase unit cell (left) of M_1_ M_2_ M_3_ M_4_AlC_3_ with elements Ti (pink), V (orange), Nb or Cr (blue), Mo (green), Al (red), and C (black). The synthesized MAX phases with layered transition metal layers are composed of four transition metal elements, with aluminum (red) and carbon (black) atomic layers in an M_4_AlC_3_ MAX structure (right). (**c**) Selective etching of the Al layers by hydrofluoric acid to synthesize multilayer high-entropy MXenes. (**d**) Delamination of multilayer MXenes is completed via organic molecule intercalants, which leads to the formation of single flakes of high-entropy MXenes TiVNbMoC3Tx and TiVCrMoC3Tx. Adapted with permission [11]. Copyright 2021, American Chemical Society.

New compositions and structures of MXenes can be obtained through additional tuning of the precursor chemistry of MXenes. The concept of high-entropy metal alloys is a material synthesis strategy that has successfully led to the creation of high-entropy MAX phases. Figure 5 depicts the schematic representation of various stages of the synthesis of two high-entropy MXenes (TiVNbMoC_3_T_x_ and TiVCrMoC_3_T_x_) from the TiVNbMoAlC_3_ and TiVCrMoAlC_3_ MAX phases [11]. High-entropy MAX phases were synthesized through the reactive sintering of elemental powders. Tetramethylammonium hydroxide (TMAOH) was used to delaminate the extracted high-entropy MXenes into single flakes of 2D MXenes. This study reports the latest modification of the fabrication pathway reported in this field [11]. Furthermore, the consequence of this synthesis route provides the enormous contribution and positive results of two interrelated aspects of “atoms and ions” and “crystal structure” while adding another milestone of tuning to this rapidly growing field. Due to its increasing popularity among new researchers, Shuck et al. published a research paper on the safety risks associated with each step of the MXene synthesis pathways and the precautions to be followed for safe, reproducible, and reliable synthesis [18]. 

## 3. Mxenes for Energy Storage Devices

As the world accelerates toward digitalization, the demand for efficient energy storage devices (ESDs) has ramped up incredibly in a short period. Batteries and supercapacitors, among all ESDs, have been identified as important contenders for efficient energy storage due to their viable electrochemical characteristics. According to reported data, batteries have a high energy density compared to supercapacitors. Supercapacitors have a high power density compared to batteries. Due to their high energy density, batteries deliver low power for long periods and are used in applications ranging from power electronics to mobility and grid storage. However, limitations in low power and the lifetime of charge-storage mechanisms in batteries constrain their expansion. The energy density of batteries is size-dependent, which limits their use in microscale and wearable devices as well. Supercapacitors are massively employed to store pulse power because of their high-rate capability and long-term cyclability. Moreover, their environmental friendliness and simple adsorption and desorption mechanism in terms of electrostatic interactions make supercapacitors a better choice than traditional batteries. In that sense, both devices have their weaknesses. For this reason, there is a great quest for sustainable, low-cost, eco-friendly alternative materials to produce high-performance batteries and supercapacitors [19]. 

MXenes have been largely investigated in energy storage applications since their discovery due to their outstanding electrical and electronic properties. The atomic thickness, crystalline nature, and layered structure of MXene facilitate a high specific surface area, a low energy barrier for electron transport, and a short ion-diffusion path. In addition, both theoretically and experimentally, it has been proved that the electronic properties of MXenes can be modified by altering their surface terminations. Thus, recent energy storage studies have been exploring new ways of using MXene and MXene-based resources to reach efficient ESDs in the next fifty years. The MXenes and MXene-based composites have been introduced into various components of ESDs, including electrodes, electrolyte, and their interface areas, in several studies, particularly in those developing new portable and flexible ESDs [20,21,22]. Therefore, this section focuses on recent studies related to the application of MXene-based materials for batteries and supercapacitors. A summary of the electrochemical performance of MXene-based materials in batteries and supercapacitors is shown in Table 1 and Table 2, respectively.

### 3.1. MXenes for Batteries

The battery manufacturing industry has grown tremendously over the decades. Recently, lead acid, lithium ion, nickel metal hydride, and nickel-cadmium batteries have gained significant attention as energy storage and supply devices in terms of usage and applications in the global battery market. However, technological advancements in terms of improved efficiency, cost-effectiveness, and product innovation have led to the proliferation of lithium-ion batteries (LIBs). In the future, LIBs are expected to infiltrate other battery applications and capture more market share due to their high energy density and low cost. However, the large number of compounds available has created a tremendous opportunity for materials scientists to discover new battery electrodes. Many studies have been conducted recently to improve the Coulombic efficiency and cyclic ability by inhibiting the Li dendrite growth of lithium-ion batteries using MXenes. Moreover, batteries incorporating MXene-based materials such as lithium-sulfur, aluminum, and zinc-ion batteries are also developing vigorously [22,23]. 

MXenes have high lithium capacity, wider interlayer spacing, a low diffusion barrier for Li ions, high electrical conductivity, and a low operating voltage (−0.2 to 0.6 V vs. Li/Li+), which can provide excellent rate performance and cycling stability [24]. Numerous MXene materials, such as Ti_3_C_2_T_x_, Mo_2_TiC_2_T_x_, Nb_2_CT_x_, V_2_CT_x_, Nb_4_C_3_T_x_, and Mo_2_CT_x_, have been examined as potential anodes for LIBs. In a study, multi-walled carbon nanotubes (MCNTs) have been uniformly grown on a Ti_3_C_2_ MXene network by an in situ facile microwave irradiation method under ambient conditions. The MCNTs@Ti_3_C_2_ composite material was assembled as the anode in an LIB to improve the cyclic stability of the LIB. The MCNTs@Ti_3_C_2_ composite exhibited high reversible capacities of 430 mAh/g at 1 A/g and 175 mAh/g at 10 A/g, which was attributed to the synergetic effects of the connective MCNT bridges, large-capacity metal/metal oxides and the fine conductive MXene matrix [25].

**Figure 6:**
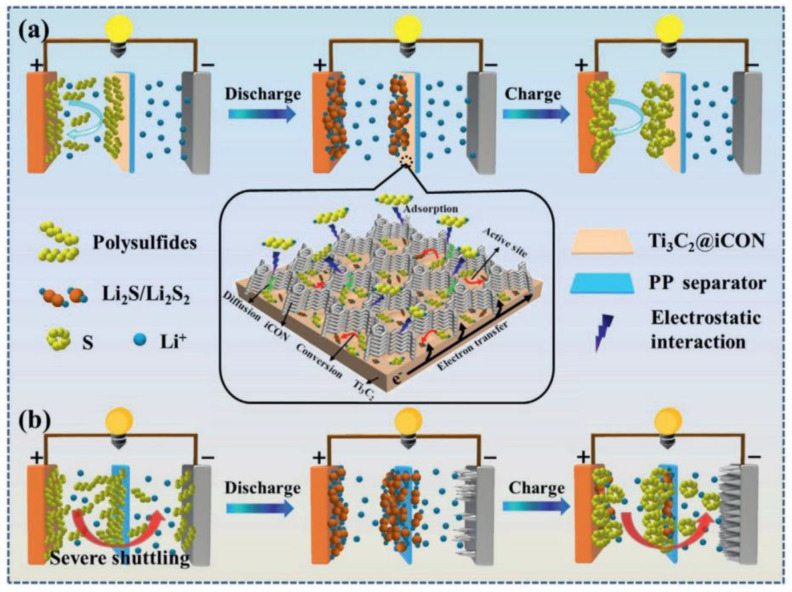
Schematic illustrations of polysulfide trapping and conversion process on (**a**) Ti_3_C_2_@iCON-PP (**b**) and Ti_3_C_2_-PP. Adapted with permission [30]. Copyright 2021, John Wiley and Sons.

With current LIB technology, lithium-sulfur batteries (LSBs) are also moving forward competitively for wider commercialization with their high specific energy. According to the literature, the theoretical capacity and specific energy density of LSBs are 1675 mAh/g and 2600 Wh/kg, respectively, which is four times higher than that of LIBs. However, their wide application is severely hampered by the low electrical conductivity of sulfur and its discharge products Li_2_S_2_/Li_2_S, as well as the migration of soluble polysulfides (Li_2_S_4_^−8^) across the separator during the charge/discharge process (“shuttle effect”) (Figure 6). Many approaches using MXene-based materials have been developed to address these issues, and it has been proven that the highly functionalized 2D surface of MXenes can effectively immobilize soluble polysulfides through metal–sulfur interactions while maintaining high electrical conductivity [26,27]. One approach is to design nanostructured cathodes by confining sulfur within conductive frameworks. MXene-based conductive composites can improve the intimate conductive contact between the insulating sulfur particles and enhance electrochemical performance. Further, the MXene component can limit the dissolution and occlusion of polysulfide intermediates through physical adsorption or chemisorption [28,29]. Another approach has been made by restricting the migration of dissolved polysulfides across separators [30]. The methods involve introducing an MXene-based functional interlayer between the separator and sulfur cathode or a coating layer on the cathode side of the separator [31]. According to reported studies, as a separator/electrode composite material, Ti_3_C_2_T_x_ MXene can provide a physical and chemical barrier to suppress polysulfide migration and remarkably increase the Coulombic efficiency and lifetime of LSBs. On the other hand, due to the excessive use of lithium metal, access to lithium metal resources, high cost, and environmental concerns are also likely to arise. 

**Figure 7:**
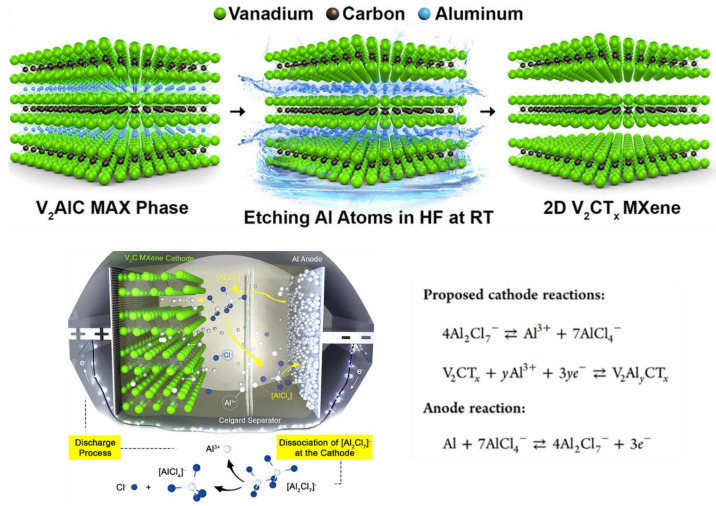
Schematic illustration of the process of selective etching used to synthesize V_2_CT_x_ MXene from the V_2_AlC MAX phase followed by interlayer expansion of ML-V_2_CT_x_ MXene via TBAOH intercalation and the proposed mechanism for an Al battery with V_2_CT_x_ MXene as the cathode during discharge in ionic liquid electrolyte. Adapted with permission [22]. Copyright 2017, American Chemical Society.

Aluminum batteries (ALBs) are another potential candidate for energy storage because Al is safer and cheaper than Li. However, (a) the high charge density of Al^3+^ cations and their strong interactions with the host lattice and (b) the low potential window limit their cyclic stability and energy density, respectively. In a study, Vahidmohammadi et al. reported that V_2_CT_x_ MXene can reversibly intercalate Al^3+^ cations into their structures to fabricate an intercalation-type cathode material for Al batteries that has excellent cycle stability and high energy density [22]. V_2_CT_x_ MXene electrodes show one of the best performances among the reported cathode materials for Al batteries so far. In this work, multilayered V_2_CT_x_ powder was synthesized by the chemical etching of Al atoms from V_2_AlC (MAX phase) by immersing an HF solution at room temperature. Thus, ALB was prepared using the layered vanadium carbide MXene (TBAOH-FL-V_2_CT_x_) as the cathode, aluminum metal as the anode, and a nonflammable aluminum chloride-based ionic liquid as the electrolyte. Figure 7 shows the schematic illustration of the procedure they followed to prepare the novel ALB with the proposed charge–discharge process at the liquid–solid interface. The TBAOH-FL-V_2_CT_x_ cathode delivered exceptionally high specific capacities of more than 300 mAh/g at a current density of 100 mA/g [22]. Thus, this research opened a new pathway to improving the performance of ALBs. Consequently, in another study, an ALB containing an MXene-based-composite cathode (F-Ti_3_C_2_T_x_@Ag) was prepared. The discharge-specific capacity of the new ALB was about 150 mAh/g after 2000 cycles at a current density of 0.5 A/g [32].

Apart from the common battery types mentioned above, rechargeable zinc-ion batteries (ZIBs) are also a battery technology that has gained significant attention. In 2020, Venkatkarthick et al. synthesized a vanadium carbide MXene-based composite with vanadium-based oxides (V_2_O_x_@V_2_CT_x_) that could serve as an efficient cathode material for an aqueous ZIB. The prepared V_2_O_x_@V_2_CT_x_ electrodes delivered an ideal rate performance with an average reversible capacity of about 304 mAh/g at a current density of 0.05 A/g [33].

### 3.2. MXenes for Supercapacitors

Supercapacitors (SCs), also known as electrochemical capacitors, are energy storage devices with high power densities, fast charge/discharge capabilities, high cyclic efficiencies, and long lives. A conventional SC consists of two solid electrodes immersed in a liquid electrolyte and divided by a membrane separator. Two electrodes are polarized by applying a voltage. According to the energy storage mechanism/potential, SCs can be subdivided into three classes: electrochemical double-layer capacitors (EDLCs), pseudocapacitors (PCs), and hybrid capacitors (HCs). EDLCs store energy based on the physical electrostatic adsorption of ions on the surface of the electrodes, while PCs use rapid electron transfer reactions occurring on the surfaces of electrodes and the electrolyte. Both energy storage mechanisms are applied in HCs. Overall, the electrode materials, electrolytes, and operation mechanisms are the main factors that determine the SC’s performance. Considering the energy density of SCs, they usually exhibit a lower value (~5 Wh/kg) compared to that of Li-ion batteries or Li-S batteries (≥50 Wh/kg) due to the low areal capacitance of the electrode materials and narrow operating potentials. The energy density equation can be expressed as W = CV^2^/7200, where W is the volumetric (or areal) energy density, C is the volumetric (or areal) capacitance, and V is the cell voltage [34]. In that sense, increasing the volumetric capacity and widening the voltage operating window are two effective ways to improve the volumetric energy density of the device. Engineered active materials with controlled nanoscale morphologies are considered to be the most effective strategy to achieve high volumetric capacity and wide operating windows in SCs because they have many reaction sites and short diffusion lengths of ions and/or electrons. Interestingly, well-designed 2D nano-MXene electrodes have shown higher supercapacitance due to their unique properties. For instance, their transition metal with variable oxidation numbers along with their unique stacked structure of MXenes makes them intrinsically conductive. Furthermore, large specific surface areas and more available redox sites in MXenes improve their electrochemical properties compared to other conventional materials, such as activated carbon, graphene, conducting polymers, and transition metal oxides, used in SCs [35].

Since the discovery of MXenes in 2011, much research work has been conducted around MXene-based SCs for energy storage purposes. Lukatskaya et al. studied the changes in the performances of Ti_3_C_2_T_x_ electrodes in SCs in acidic and basic electrolytes. They found that binder-free Ti_3_C_2_T_x_ paper exhibited 442 F/cm^3^ of volumetric capacitance at 2 mV/s in a KOH electrolyte, while a Ti_3_C_2_T_x_ clay electrode showed a higher amount of capacitance (900 F/cm^3^) at the same scan rate in an H_2_SO_4_ electrolyte [36,37]. These results lead to motivating innovations. In MXene electrodes, interlayer spacers such as metal oxides, carbon nanotubes, and reduced graphene oxides are used to increase the gap of the MXene nanosheets. Although these prevent the attachment of individual layers, on the other hand, they contribute to increasing the performance of SCs. Herein also, the electrochemical performance of the MXene composite is much higher than that of pristine MXene electrodes. Geng et al. discovered that a highly flexible and conductive composite with a better performance of the composite was shown by Ti_3_C_2_T_x_/MnO_2_ [38]. Another study created abundant channels in a highly conductive MXene network to accommodate fast electron transport and ion diffusion kinetics while maintaining a high electrode tap density. The fabricated supercapacitor was highly compact, with flexible MXene hybrid paper intercalated by Fe_2_O_3_ nanoparticles (Fe_2_O_3_ NPs@MX). The uniformly dispersed Fe_2_O_3_ NPs effectively expanded the interlayer spacing of MXene nanoflakes, shortened ion diffusion paths, and exposed more active sites. At the same time, the conductive MXene skeleton appropriately suppressed the volume expansion of Fe_2_O_3_ NPs during redox reactions. Thus, the synergistic effect of MXene and Fe_2_O_3_ NPs resulted in an extremely high volumetric capacitance of 2607 F/cm^3^ (584 F/g) and excellent cycling performance [39]. Those examples confirmed that composites of metal oxides with MXenes provide superior pseudocapacitive performance in SCs. Apart from titanium carbide MXene, a polyaniline and V_2_C MXene composite was found for the first time by Wang et al. Using this material combination, they were able to synthesize SCs with a high-density and high-sensitivity ammonia sensor [40].

**Table 1:** Summary of electrochemical performance of MXene-based materials in batteries.

Type of Battery	MXene-Based Material	Function	Capacity	Rate	Remarks	Ref.
Zinc-ion batteries	V_2_O_x_@V_2_CT_x_	Cathode	304 mAh/g	0.05–2 mA/g	Retained battery capacity constant for 200 cycles.	[33]
	Ti_3_C_2_Br_2_	Cathode	97.6 mAh/g	0.5 A/g	Retained 80% of battery capacity (at 4 A/g) for 1000 cycles.	[41]
Aluminum batteries	TBAOH-FL-V_2_CT_x_	Cathode	>300 mAh/g	150 mA/g	Maintained a capacity of about 150 mAh/g with a Coulombic efficiency of 95% at a high current density of 300 mA/g.	[22]
Lithium-ion batteries	Ti_3_C_2_T_x_	Anode	>200 mAh/g	0.1 C	Retained 80% of battery capacity (1 C) for 500 cycles.	[24]
	CNTs@Ti_3_C_2_	Anode	430 mAh/g	1 mA/g	Dropped and regained after 500 cycles.	[25]
	TiO_2_/Ti_3_C_2_T_x_	Anode	267 mAh/g	0.2 mA/g	Dropped and regained after 2000 cycles.	[42]
	Ti_3_C_2_T_x_	Anode	1 mAh/cm^2^	1.0 mA/cm^2^	Retained 98.8% of battery capacity for 450 cycles.	[43]
	Ti_3_C_2_T_x_/NiCo_2_O_4_	Anode	1330 mAh/g	0.1 C	Retained after 100 cycles.	[44]
Lithium−sulfur batteries	Ti_3_C_2_T_x_	Separator	860.7 mAh/g	0.2 C	Constant up to 30 cycles.	[26]
	Ti_3_C_2_	Separator	1201 mAh/cm^3^	0.1 C	Constant up to 2000 cycles at 2C.	[30]
	Co-CNT@MXene/S	Cathode	900 mAh/g	1 C	Constant up to 840 cycles.	[28]
	TiO_2_/H–Ti_3_C_2_T_x_	Cathode	740 mAh/g	2C	Retained 81% of battery capacity (at 1 C) for 500 cycles.	[45]

**Table 2:** Summary of electrochemical performance of MXene-based materials in supercapacitors.

Electrode	Electrolyte	Capacitance	Stability	Ref.
Ti_3_C_2_T_x_	1 M H_2_SO_4_	910 F/cm^3^ at 2 mV/s	100% after 10,000 cycles	[15]
Ti_3_C_2_T_x_-Li film	1 M H_2_SO_4_	892 F/cm^3^ at 2 mV/s	100% after 10,000 cycles	[46]
MXene/rHGO	3 M H_2_SO_4_	1445 F/cm^3^ at 2 mV/s	93% after 10,000 cycles	[47]
Ti_3_C_2_T_x_/PANI	1 M H_2_SO_4_	272.5 F/g at 1 A/g	71.4% after 4000 cycles	[48]
Mo_1.33_C MXene/PEDOT:PSS	1 M H_2_SO_4_	1310 F/cm^3^ at 2 mV/s	90% after 10,000 cycles	[49]
Fe_2_O_3_ NPs@ Ti_3_C_2_T_x_	3 M H_2_SO_4_	2607 F/cm^3^ at 1 mV/s	121% after 13,000 cycles	[39]
EE- Ti_3_C_2_T_x_ film	3 M H_2_SO_4_	1160 F/cm^3^ at 1 mV/s	100% after 5000 cycles	[50]
Ti_3_C_2_T_x_/AC/TEAPW12	1 M TEABF4 in acetonitrile	76 F/g at 1 mV/s	102% after 10,000 cycles	[51]
Ti_3_C_2_T_x_/CMC	Polyvinyl alcohol/LiCl hydrogel	113.13 mF/cm^2^ at 0.2 mA/cm^2^	97.2% after 5000 cycles	[52]
V_2_NT_x_	3.5 M KOH	112.8 F/g at 1.85 A/g	96% after 10,000 cycles	[53]

## 4. MXene-Based Electrocatalysts

The global demand for highly efficient energy conversion devices/pathways such as fuel cells, metal-air batteries, and electrochemical water splitting is boosting the development of electrocatalysts for oxygen reduction reactions (ORR), oxygen evolution reactions (OER), and hydrogen evolution reactions (HER). Often, a noble-metal-based electrocatalyst (Pt, PtIr, IrO_2_, RuO_2_, etc.) is used for these reactions to proceed at a significant rate. The scarcity and high cost of these noble metals have hindered their large-scale use and hindered their commercialization [54,55].

Electrochemical water splitting for hydrogen/oxygen generation is a strong platform for sustainable clean energy production. Water reacts to form oxygen and protons at the anode (OER) and hydrogen at the cathode (HER). OER is highly pH dependent, and strongly different pathways are observed in acidic or alkaline conditions. The two types of equilibrium half-cell reactions in OER with electron potentials (at 1 atm and 25 ℃) are given in the following equations [56]. Kinetically, this process occurs through multi-step reactions with only one electron transfer in each step, and each of these steps requires specific activation energy. Therefore, it makes OER kinetics very slow and recreates a large overpotential (Equations 4 and 5). In addition, the partial oxidation of catalysts during the electrochemical reaction is practically unavoidable. Therefore, the main challenge for a suitable OER process is to develop catalysts that combine high activity and good stability. Furthermore, OER has attracted increasing attention in the past few years because of its key role in rechargeable metal-air batteries. As illustrated by Equations (6) and (7), the HER reaction proceeds through the reduction of protons or water molecules to a hydrogen gas [57]. The standard reduction potential of the HER is defined as 0 V relative to a standard hydrogen electrode at pH 5. However, all electrochemical processes must overcome a certain overpotential. Therefore, electrocatalysts are needed to lower the HER overpotential and promote the reaction kinetics as required. Hence, OER is the limiting reaction of water splitting due to its relatively large overpotential [58,59].

**Oxygen evolution reaction**

4OH^−^ ↔ 2H_2_O_(l)_ + O_2(g)_ + 4e^−^    ⁰E_a_ = 0.404 V    (in alkaline solution) (4)

2H_2_O_(l)_ ↔ 4H^+^_(l)_ + O_2(g)_ + 4e^−^    ⁰E_a_ = 1.230 V    (in acidic/neutral solution) (5)

**Hydrogen evolution reaction**

2H_2_O_(l)_ + 2e^−^ ↔ H_2(g)_ + 2OH^−^_(aq)_    (in alkaline solution) (6)

2H^+^_(aq)_ + 2e^−^ ↔ H_2(g)_    (in acidic/neutral solution) (7)

In many cases, MXene nanomaterials have attracted considerable attention among researchers due to their good metallic conductivity, low weight, high surface area, and durability. In this sense, MXene-based materials can also be successfully used as electrocatalysts. Moreover, it is feasible to optimize the specific electroactive sites of MXenes by controlling the surface chemistry of MXene during the selective etching process. The delamination and exfoliation of MXenes into a single or few layers using intercalants expose a large surface area for catalysis. Mxene hybrid and composites exhibit significantly enhanced catalytic activities and stability compared to pristine MXene due to synergistic coupling between MXenes and their secondary materials. Currently, numerous organic and inorganic materials such as TM carbides, TM phosphides [60], TM chalcogenides, TM oxides [61], layered double hydroxides, metal-organic frameworks [62,63,64], graphitic carbon nitride [65], carbon nanotubes [66], quantum dots, metallic alloys, polymers, etc. have been combined with MXenes to form MXene hybrids/composites [55,61,67].

**Figure 8:**
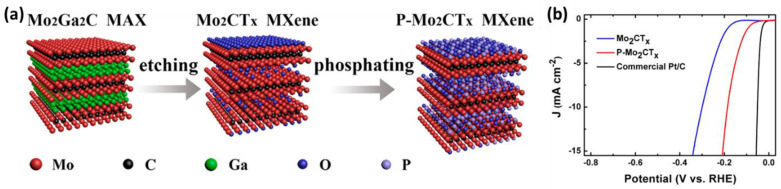
(**a**) Schematic illustration of the preparation of Mo_2_CT_x_ and P−Mo_2_CT_x_ and (**b**) HER polarization curves of Mo_2_CT_x_, P−Mo_2_CT_x_, and commercial Pt/C (20 wt % Pt). Adapted with permission [68]. Copyright 2018, American Chemical Society.

For example, P and O-doped Mo_2_CT_x_ MXenes were able to improve the HER electrocatalytic performance compared with pristine Mo_2_CT_x_ MXenes, with a dramatic decrease in overpotential (more than 100 mV at 10 mA/cm^2^). The enhanced catalytic performance of phosphorized Mo_2_CT_x_ MXenes can be attributed to: (a) increased active sites due to the expanded interlayer distance, (b) the formation of new P and O active sites for hydrogen absorption, and (c) the improved metallic band structure of Mo_2_CT_x_ due to P incorporation. The synthesis of Mo_2_CT_x_ and P-Mo_2_CT_x_ is illustrated in Figure 8. The Mo_2_CT_x_ MXenes were prepared by selectively etching the Ga layer from Mo_2_Ga_2_C MAX powders in a mixed solution of LiF and HCl. Mo_2_CT_x_ Mxenes were then subjected to a simple phosphorization to prepare P−Mo_2_CT_x_ [68].

**Figure 9:**
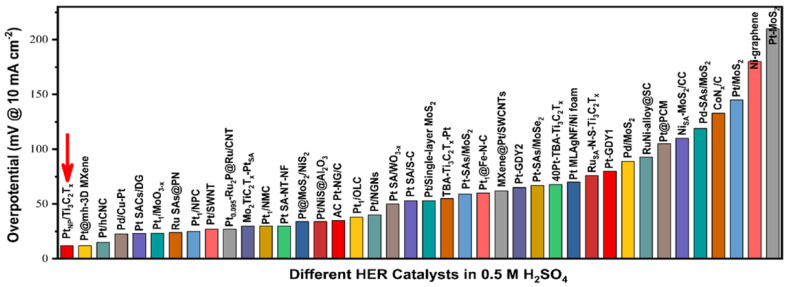
Overpotential comparison of Pt_NP_/Ti_3_C_2_T_x_ with that of the previously reported Pt-based HER electrocatalyst in an acidic electrolyte. Adapted with permission [69]. Copyright 2022, American Chemical Society.

Furthermore, a binder-free Ti_3_C_2_T_x_ Mxene-supported low-Pt loading electrocatalyst (Pt_NP_/Ti_3_C_2_T_x_) was prepared to catalyze HER by Jian et al. PtNP/Ti_3_C_2_T_x_ showed a low overpotential of 12 mV at a current density of 10 mA/cm^2^ in an acidic medium. This value is comparable to other noble metal-based HER electrocatalysts reported in recent literature under the same conditions (Figure 9). Thus, this study opens a new and convenient avenue for the preparation of highly efficient binder-free Mxene-supported metal electrocatalysts [69].

**Figure 10:**
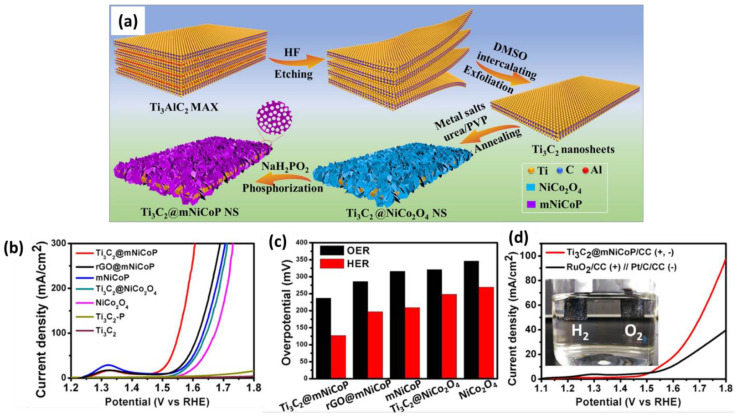
(**a**) Schematic illustration of the synthesis of hierarchical Ti_3_C_2_@mNiCoP NS and electrochemical test results, including a comparison of (**b**) polarization curves, (**c**) overpotentials, and (**d**) a photo of a water-splitting cell. Adapted with permission [70]. Copyright 2020, American Chemical Society.

Theoretical and experimental studies proved that transition-metal phosphides, especially Co−P, Ni−P, or their bimetallic phosphide, can accelerate the HER and OER [60,70]. Since transition-metal phosphides usually possess poor conductivity, conductive carbon supports are integrated with metal phosphide catalysts for efficient electrocatalysis. A group of scientists then constructed well-defined hierarchical 2D sandwich structures for electrocatalytic water splitting by combining exfoliated Ti_3_C_2_ Mxene (as a substrate) with mesoporous nickel cobalt phosphide nanosheets (mNiCoP NS). Taking advantage of its unique properties, including its good conductivity, high surface area (143.5 m^2^/g), abundant exposed active sites, and good structural/chemical stability, Ti_3_C_2_@mNiCoP NS exhibits superior overall water splitting performance over its building-block counterparts (Figure 10) [70]. These results highly suggest that MXene-based materials can be used to develop low-cost and robust electrocatalysts with intrinsic active sites capable of catalyzing HER and OER processes. ORR is the key reaction at the cathode of proton exchange membrane fuel cells, direct methanol fuel cells, and metal-air batteries. Similar to OER, the ORR also suffers from sluggish reaction kinetics, resulting in a decreased power density and a large overpotential. This obstacle can be effectively addressed by preparing MXene-based electrocatalysts [71].

## 5. MXene-Based Sensors

Excellent surface chemistry and electroconductivity are the required primary properties of a material that is used for sensor fabrication. The characteristic features of MXenes and MXene-based materials, including their higher sensitivity, linear responses to signals, low hysteresis, ability to quickly recover for repetitious use, and low fabrication cost, have indicated the use of these materials for a broad range of sensor fabrications, for instance, toxic compound identification, human health monitoring, humidity and gas sensing, etc. Secondary nanoparticles, including graphene oxide (2D), carbon nanotubes (1D), and silver nanoparticles (0D), have been mixed with MXene to create better MXene-based heterostructures [72,73]. Electrostatic attractions play an important role during the mixing of 2D, 1D, and 0D secondary nanoparticles with MXene. 2D + 2D MXene-based heterostructures are widely used for sensing applications, 2D + 1D MXene-based heterostructures are used for catalysis, and 2D + 0D MXene-based heterostructures are used for energy storage and conversion.

The sensitivity performance of MXene materials depends on the types of functional groups on the surface and their concentration. For instance, hydroxyl-terminated MXene nanocomposites exhibited better sensing performance for ethanol detection, and oxygen-terminated MXene surfaces exhibited excellent performance for ammonia sensing. Polyaniline/MXene (PANI/Ti_3_C_2_T_x_) nanocomposites are used as a high-sensing material for room-temperature gas detection [74,75,76]. A PANI/Ti_3_C_2_T_x_ nanocomposite was synthesized using in situ polymerization at low temperatures to securely anchor PANI nanoparticles on the MXene nanosheets. PANI nanoparticles prevented the staking of Ti_3_C_2_T_x_ MXene nanosheets, and different functional groups (-OH, -F, -O) in MXene nanosheets provide an absorption surface for gas molecules. The PANI/Ti_3_C_2_T_x_ nanocomposite exhibited high ethanol sensitivity (41.1%, 200 ppm) and faster response/recovery time (0.4/0.5 S) at room temperature. Moreover, stable sensitivity, mechanical stability, and ultrafast response rates were shown by PANI/Ti_3_C_2_T_x_-based flexible sensors after continuous bending from 0° to 120° [74].

The enzyme immobilization potential of MXene is another characteristic property that is highly effective for biosensors, and MXene provides a microenvironment for an enzyme to maintain its stability and activity [77]. Ti_3_C_2_ Mxene mixed with the enzyme acetylcholinesterase (AChE) and chitosan to fabricate nanocomposite biosensors can be appropriate for the detection of organophosphate pesticides (Ops) in water. A cshitosan/Mxene nanocomposite exhibited a low detection limit (0.3 × 10^−14^) with a linear dynamic range. Therefore, that sensing material was successfully applied to detect toxic pesticides in water, and it could be used to test if food products are contaminated with Ops. The concentration of chitosan provides a resistance to the chitosan/Mxene nanocomposite. To avoid that condition, a high concentration of HF etching was used as an effective method during the synthesis of the chitosan/Mxene nanocomposite to immobilize enzymes in chitosan because it provides a high surface area without increasing the resistivity [73]. Additionally, a hemoglobin-immobilized Nafion/MXene composite exhibited a wide linear range of detection from 0.5 µM to 11,800 µM with a low detection limit for nitrite ions [78].

Attention toward wearable pressure sensors has grown due to broad applications, including the physiological monitoring of body organs, human-machine interfaces, and e-skin development. Secondary nanoparticle-embedded MXene materials and MXene- based piezoresistive sensing materials are used for pressure sensor fabrications due to their high sensitivity and excellent flexibility [79,80]. However, challenges, including limited deformability and biofunctionality for external stimuli, large hysteresis, and long response time, have mainly limited the applications of these pressure sensors in different fields. To overcome these challenges, scientists have searched for functional materials or structures that can be embedded with MXene materials to convert pressure differences into an electrical signal. Moreover, scientists have observed that naturally evolved 3D architectural biological materials have distinctive properties that are sensitive to environmental changes [81].

Interlocked microstructures located between dermal and epidermal layers in the human skin act as a sensing area, and identified pressure stimuli can effectively transduce to cutaneous receptors located beneath the dermal layer. Imitating this phenomenon, scientists introduced biomimetic interlocked structures by assembling natural microcapsules in 2D Ti_3_C_2_ MXene nanosheets. Biomimetic interlocked structures exhibit enhanced mechanical stimulus sensing performance due to their higher deformability. The pressure sensitivity of biomimetic interlocked structures was improved by over 9.4 times compared to a planar Ti_3_C_2_-based flexible film without biomimetic interlocked structures. Moreover, their low detection limit, fast response, and excellent mechanical reversibility confirmed the superiority of the pressure sensor and opened doors for applications in various fields [81].

The surface modification of MXene and its self-healing properties are essential to improve the affinity between the polymeric phase and MXene. The performance stability of electronic sensors decays due to inevitable cracks and scratches during continuous deformation. To avoid this problem, scientists have developed sensing materials with self-healing properties to enhance their reliability and lifespan. As the brittle and rigid structure of MXene is susceptible to mechanical deformation and limits its applications in electronics, the incorporation of MXene with soft polymers has been pursued to improve its sensing and mechanical properties. Notwithstanding, MXene/rubber-based sensor materials have experienced poor performance stability and response reliability due to less interfacial interactions between the polymer matrix and the MXene surface. Therefore, the MXene nanosheet surface was modified through an esterification reaction. A nanostructured Ti_3_C_2_ MXenes/rubber-based supramolecular elastomer (NMSE) was used as a robust, self-healing, flexible sensor for electronics. The preparation of the NMSE was inspired by amino acid interactions in proteins. A polypeptide, which is formed through different interactions between amino acids, including hydrogen bonding, van der Waals interactions, and hydrophobic interactions, can self-assemble to form a large protein. In addition, supramolecular interfacial interactions formed by amino acids provide a dynamic bonding interface to create new bonds for self-healing materials.

**Figure 11:**
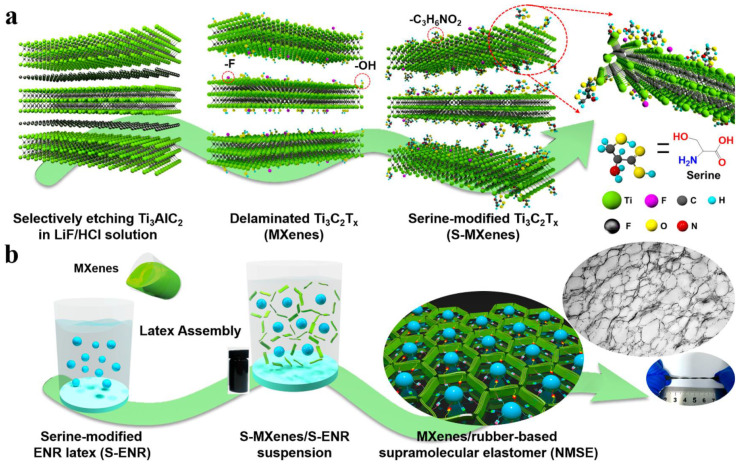
The main preparation process for NMSE. (**a**) Surface modification of MXene nanosheets by serine via the esterification reaction. (**b**) Construction of a nanostructured MXene network in NMSE via the latex assembly method. Adapted with permission [82]. Copyright 2020, American Chemical Society.

The MXene nanosheet surface was modified using an esterification reaction between hydroxyl groups and carboxyl groups on serine. Afterward, serine-grafted epoxidized natural rubber (S-ENR), which formed after reacting ENR’s epoxy group and amino groups of serine molecules, was merged with surface-modified MXene by the latex assembly process. Finally, surface-modified MXene nanosheets moved to the S-ENP latex microspheres and formed a segregated 3D conductive network (Figure 11). The formed 3D conductive network facilitated the formation of supramolecular hydrogen interactions between the unreacted hydroxy and carboxyl groups of S-ENR and the amino and hydroxyl groups of surface-modified MXene. Supramolecular hydrogen bonding between the esterified MXene surface and the elastomer chain facilitated the self-healing property of NMSE at room temperature. Moreover, hydrogen bonding decreased the percolation thresholds of MXene due to the formation of a segregated 3D conductive network and improved the mechanical property and twist ability of the sensor [82].

Environmental monitoring and non-invasive epidermal sensing bring forward the requirement for fast humidity sensors. However, the fabrication of fast humidity sensors is challenging because the response of the sensor depends on the sensing method, the diffusion of water, and water sorption in the sensing material. MXene/polyelectrolyte multilayer forms are successfully used as a sensing material to detect humidity. When humidity changes (water molecules intercalate with multilayer forms), the thickness and sheet-to-sheet distance of MXene/polyelectrolyte multilayers change, and these changes cause changes in the tunneling resistance between MXene nanosheets. Hydrophilicity and higher conductivity nature are directed to the use of MXene as a sensing material for humidity sensors. The layer-by-layer method was used to fabricate MXene/polyelectrolyte multilayers, and higher response and recovery times were achieved [83].

## 6. Other Emerging Applications of MXene

Recently, many research works have explored the application of MXenes in electromagnetic interference shielding (EMI), biomedical applications, flexible and wearable devices, and membranes. In particular, the discovery of MXenes has revolutionized these applications. In this section, recent progress in these fields regarding MXene-based materials is summarized.

Mxene’s mechanical flexibility, hydrophilicity, and biocompatibility contribute to its use as a material for biomedical applications, including in tissue engineering, drug delivery [84,85], bioimaging, sensors, and as an antibacterial [86]. Regarding tissue engineering, MXene can be used as a material mainly for bone tissue engineering [87,88], myocardial tissue engineering [89], and nerve tissue engineering [90]. MXene/PLLa-PHA composite nanofibers prepared through electrospinning and the doping strategy were used as a smart biomaterial for cell cultures. MXene/PLLa-PHA nanofibers exhibited improved hydrophilicity due to the presence of hydrophilic groups. Those functional groups and nanosurfaces created an excellent microenvironment for bone marrow-derived mesenchymal stem cell growth. Cell testing of an MXene composite nanofiber confirmed the presence of good biocompatibility and the excellent improved cellular activity of the MXene composite nanofiber [91]. Controlled and slow-release drugs are a newly emerging research area in cancer chemotherapy because controlled drug release can minimize the cytotoxic effect of the most common anticancer drugs, including cisplatin, paclitaxel, etc. MXene-based materials have been used as drug carriers due to having functional group-rich surfaces, biocompatibility, and planar structures. Surface-modified nanosized Ti_3_C_2_ with a negatively charged surface of MXene was used to deliver cationic anticancer drugs because nanosized Ti_3_C_2_ has an enhanced permeability and retention effect and can accumulate at the tumor site. Tumor sites have lower pH than other normal tissues, and this acts as a driving force to break the electrostatic interaction between the drug and MXene [92]. Moreover, MXene is used as a material for photothermal therapy (PTT), photodynamic therapy (PDT), and thermodynamic therapy to treat cancer cells. PTT is a minimally invasive treatment method that can remove cancer cells by absorbing near-infrared radiation at the site of the cancer cells and converting it into heat. The higher photothermal conversion efficiency and larger surface area of MXene mainly indicate its use as a material for PTT. PDT is a non-invasive and effective treatment strategy. Due to the optical and electrical properties of MXene, it can be used as a photosensitizer in PDT [86].

EMI occurs when an electronic device is exposed to an electromagnetic field. With the ever-increasing use of more complex, sophisticated, and miniaturized electronic devices, EMI can create detrimental effects on the performance of that device. In this regard, to protect electrical and electronic equipment from EMI, it is necessary to develop an efficient shielding material with minimal transmittance. MXenes and MXene-based composites have a more excellent EMI shielding effect than conventional materials; for instance, 2D C-based materials (e.g., expanded graphite, graphene, reduced graphene oxide), metals (e.g., silver, aluminum, copper), and metallic fillers [93]. Three MXene films have been extensively investigated for EMI shielding applications, including single-metal Ti_3_C_2_T_x_, ordered double-metal Mo_2_TiC_2_T_x_, and Mo_2_Ti_2_C_3_T_x_ MXene. Their excellent capping performance, outstanding metal conductivity, low density, large specific surface area, tunable surface chemistry, and solution processing capability drive their use. Meanwhile, Ti_3_C_2_T_x_ outperformed Mo_2_TiC_2_T_x_ and Mo_2_Ti_2_C_3_T_x_. Many MXene composites and hybrids with other conducting or magnetic ingredients have been explored to further improve the inherent EMI shielding properties of MXenes [94].

Wearable and flexible devices (WFDs) can be applied to almost every critical aspect of our lives, including physical activity monitoring, health monitoring, treatment referral, communication, etc. Thus, many studies have been conducted frequently on the preparation, design, and application of WFDs. Most importantly, a WFD should be flexible, lightweight, highly durable, skin-friendly, and mechanically robust. Various types of WFDs, such as supercapacitors, electronics, sensors, and EMI shields, have been researched over the years to combine and improve the above-mentioned features. MXene is considered a favorable material for hybrid applications due to its unique properties, such as its outstanding electrical conductivity, large specific surface area, distinctive layered structure, excellent dispersibility in aqueous solutions, and abundant, tunable terminal groups. However, its poor mechanical properties, easy restacking, relatively small lateral size, and poor stability in an oxygen atmosphere greatly limit its usage as pristine MXenes. Interestingly, the desired characteristics can be achieved by combining MXene into composites with other materials [95,96]. As an outstanding representative of the MXene family, Ti_3_C_2_T_x_ has broad prospects for WFDs. Its unique and controllable surface chemical structure, high metallic electrical conductivity and double layer capacity, excellent biocompatibility, and large specific surface area help to suit wearable and flexible applications [97].

Owing to the hydrophilic behavior, high adsorption capacities, and tunable surface chemistry of MXene, it has been used as a membrane for water purification and in the remediation of environmental pollution, such as in the adsorption/photodecomposition of dyes and the adsorption of heavy metals in wastewater. For example, Shahzad et al. investigated the adsorption and removal of copper (Cu), which is in an aqueous medium, using delaminated Ti_3_C_2_T_x_ MXene nanosheets as the membrane material. They showed that delimitated Ti_3_C_2_T_x_ can uptake Cu with a 78.45 mg/g adsorption capacity. This result was 2.7 times higher than commercially available activated carbon [98]. Nanofiltration (NF) membranes have attracted increasing attention in mono/divalent ion separation. The most advanced NF membranes are prepared via the interfacial polymerization of polyamide on a porous support layer. The interfacial polyamide layer controls the physicochemical properties and separation performance of the NF membranes. Embedding MXenes into polyamide thin-film membranes is an effective modification technology to enhance membrane performance. In 2021, a group of scientists synthesized polyamide nanocomposite NF membranes by ultrasonically dispersing Ti_3_C_2_T_x_ MXene in an organic phase (n-hexane) for desalination. The organic phase-enabled MXene nanosheets were deposited on the membrane surface, which directly corresponded to enhancing the negative charge on the surface (due to the abundant oxygen-containing and fluorine-containing surface functional groups). MXene embedded in the organic phase increases the crosslinking degree of polyamide and lowers the effective pore size of the membrane. Therefore, Ti_3_C_2_T_x_ MXene shows potential advantages in improving the desalination performance of NF membranes. This recent finding could provide theoretical guidance for future research in this field [99].

## 7. Perspectives of MXene-Based Nanomaterials

Anasori and Gogotsi confirmed that the use of MXenes in biomedical, mechanical, electronic, and electromagnetic fields greatly expands this period from an application perspective. The utilization of MXene for new applications is increasing day by day beyond our expectations. One example is the use of MXenes to produce lubricants to reduce friction and wear. In addition, new computational quantum mechanical studies are projected to improve the electronic and magnetic properties of MXenes (especially rare-earth metal carbides) needed to fabricate 2D magnets. As a result of these theoretical and experimental discoveries, many new compounds belonging to the MXene subfamily of high-entropy 2D metal carbides will emerge in the coming years. With the most recent discovery of oxycarbide MXenes in late 2022, researchers confirmed the existence of a new subfamily of MXenes [100]. Successful applications of this subclass of compounds are still being researched. The entire scientific world is waiting to see how this “game-changing compound in the world of materials” will contribute to the development of new technologies. The details provided in this review will be very beneficial in gaining a comprehensive knowledge of employing MXene in multifunctional applications.

## 8. Conclusions

This review emphasised MXene’s adaptability in electrochemical energy storage devices, electrocatalysis, sensors, electromagnetic shielding, biomedical applications, membranes, and flexible and wearable devices. The unique compositional variables of MXenes are most helpful in achieving high performance in the aforementioned applications due to their various combinations and surface terminations. Typically, the top-down separation of stacked MXene sheets from the MAX phase is the most typical synthesis method, and the MAX phase, etching procedure, functional groups (T_x_), intercalants, and delamination process affect MXene materials. Compared to pristine MXenes, MXene composites/hybrids have excellent capacities, superior cyclability, and excellent cyclic stability due to the large specific surface area of carbon-based materials and the electroactive sites of MXene in the composite, which greatly enhance electronic/ion transport capabilities and supercapacitance with the synergistic contributions of both double layers and Faradaic capacitances. Moreover, MXene-based materials are also effective electrocatalysts for HER, OER, and ORR; for example, the Pt@Ti_3_C_2_T_x_ MXenes demonstrated outstanding HER activity (below 50 mV@10 mA/cm^2^). On the other hand, MXenes’ mechanical flexibility, hydrophilicity, higher photothermal conversion efficiency, and biocompatibility properties point to the use MXenes for biomedical applications, including as tissue engineering, sensors, therapeutics, and drug delivery systems. Moreover, Mxene-based cancer therapies, including controlled drug release, PTT, and PDT, have become hot topics in the biomedical field due to their excellent properties. Moreover, single-metal Ti_3_C_2_T_x_, ordered double-metal Mo_2_TiC_2_T_x_, and Mo_2_Ti_2_C_3_T_x_ MXene are mainly used for EMI shielding due to their unique properties compared with other conventional materials. Despite the fact that MXenes have shown excellent performance in a variety of applications, several considerations, such as (i) understanding the structure-property relationships, (ii) the combination of computational, machine learning, and experimental studies, and (iii) the utilization of in situ SEM/TEM techniques, should be taken into account for the continued development of MXene-based materials.

## Data Availability

Not applicable.
